# 

**Published:** 1897-11

**Authors:** 


					﻿Society Notes.
Cameron, Texas, October 26th, 1897.
Dear Doctor:—The Third Semi-Annual Meeting of the South
Texas Medical Association will meet in Beaumont, Texas, De-
cember 28th, 1897.
We cordially invite you to be present, and take part in this
meeting, making it the grandest meeting in the history of the
Association.
If you desire to read a paper, notify the Secretary on or be-
fore November 15th, in order that the programs may be printed
early.
The physicians of Beaumont are enthusiastic over this meet-
ing, and with Dr. Price as Chairman of the Arrangement Com-
mittee, success is assured.
Do not forget the date, December 28th, 1897.
Bat Smith, M. D., President,
E. S. Ferguson, M. D., Secretary, Wharton, Texas.
Cameron, Texas.
Brazos Valley Medical Association.
Dear Doctor:—The Brazos Valley Medical Association is
nearing its Fourth Semi-Annual meeting, which convenes at
Navasota the second Tuesday and Wednesday in November.
We can point with pardonable pride to the achievements of the
Association during its first year of existence, and now let me
kindly ^insist that you lay aside the busy cares of life for a time
and meet with us, remembering, if we raise and maintain the
standard of true medicine in Texas, it will require a united ef-
fort on the part of her votaries.
Ample means are being provided for our comfort, and the
welfare of the local Association by the local brethren, and it will
be a season of refreshment and learning to all who attend.
Fraternally yours,
G. M. Abney, President.
PROGRAMME.
1.	Paper, Placenta Previa—Dr. J. 0. Hollman, Franklin.
Discussion—Dr.- B. F. Watkins, Bryan; Dr. A. J. Elisey,
Lilac.
2.	Paper, Pneumonia—Dr. I. P. Sessions, Rockdale. Dis-
cussion—Dr. J. P. Oliver, Caldwell; Dr. T. J. Curry, Whee-
lock.
3.	Paper, The Use of Cocaine as an Anaesthetic—Dr. H. W.
Cummings, Hearne. Discussion—Dr. R. Carrol, Calvert; Dr.
M. L. Langford, Bailyville.
4.	Paper, Intestinal Anastimosis—Dr. E. N. Shaw, Cam-
eron. Discussion—Dr. J. A. Bodine, San Antonio; Dr. Geo.
R. Tabor, Bryan.
5.	Paper, Uterine Displacements—Dr. E. A. Harris, Nava-
sota. Discussion—Dr. H. L. Fountain, Bryan; Dr. A. Kobro,
Taylor.
6.	Paper, to be supplied—Dr. J. A. Bodine, San Antonio.
Discussion—Dr. W. R. Vaughn, NesbP; Dr. A. C. Scott, Tem-
ple.
7.	Paper, Indolent Ulcers—Dr. Daniel Parker, Calvert.
Discussion—Dr. W. W. Greer, Cameron; Dr. E. Brittain, Bre-
mond.
8.	Paper, Typhoid Fever—Dr. E. A. Thompson, Navasota.
Discussion—Dr. J. M. Nicks, Stone City; Dr. J. P. Barnett,
Navasota.
9.	Paper, The Hot Permanganate of Potash, Irrigation in the
Treatment of Gonorrhoea; Dr. R. W. Nobles, Temple. Discus-
sion—Dr. W. H. Harrison, Navasota; Dr. Moore, Buckholts.
10.	Paper, Formaldehyde, its Uses—Dr. A. C. Scott, Tem-
ple. Discussion—Dr. A. L. Hathcock, Palestine; Dr. W. T.
Wilson, Navasota.
11.	Paper, to be supplied—Dr. E. N. Davis, Houston. Dis-
cussion—Dr. O. L. Norsworthy, Houston; Dr. J. C. Vannuys,
Franklin.
12.	Paper, Report of a Laparotomy for Obstruction of the
Intestines—Dr. F. R. Collard, Wheelock. Discussion—Dr. J.
M. Gray, Milano; Dr. J. A. Boyd, Thorndale.
13.	Tuberculosis of the Joints and Bones. Dr. D. L. Pee-
ples, Navasota. Discussion—Dr. T. A. Pope, Cameron; Dr. A.
C. Scott, Temple.
Voluntary papers and report of cases.
				

